# Supplementary material for: Influencing Cardiovascular Outcomes through Heart Rate Variability Modulation: A Systematic Review
Source: Diagnostics (Basel). 2021 Nov 25;11(12):2198. doi: 10.3390/diagnostics11122198 (PMC8700170; doi:10.3390/diagnostics11122198)
Supplement: Supplementary file 1 [file diagnostics-11-02198-s001.zip › diagnostics-1456342-supplementary/Table S1 Databases and search strategy.pdf]

Table S1. Databases and search strategies used in present systematic review

Database	Coverage	Search run	Records
**MEDLINE**	1946 to present	"(heart rate variability) AND ((biofeedback) OR (resonance frequency breathing))"	97
		"(heart rate variability) AND ((biofeedback) OR (resonance frequency breathing)) AND ((heart failure) OR (coronary artery disease) OR (arterial hypertension))"	17
		"(heart rate variability) AND ((biofeedback) OR (resonance frequency breathing)) AND ((cardiovascular events) OR (mortality))"	5
			Total records = 119
**Embase**	1966 to present	"(heart rate variability) AND ((biofeedback) OR (resonance frequency breathing))"	34
		"(heart rate variability) AND ((biofeedback) OR (resonance frequency breathing)) AND ((heart failure) OR (coronary artery disease) OR (arterial hypertension))"	5
		"(heart rate variability) AND ((biofeedback) OR (resonance frequency breathing)) AND ((cardiovascular events) OR (mortality))"	6
			Total records = 45
**Cochrane library**	1967 to present	"(heart rate variability) AND ((biofeedback) OR (resonance frequency breathing))"	244
		"(heart rate variability) AND ((biofeedback) OR (resonance frequency breathing)) AND ((heart failure) OR (coronary artery disease) OR (arterial hypertension))"	23
		"(heart rate variability) AND ((biofeedback) OR (resonance frequency breathing)) AND ((cardiovascular events) OR (mortality))"	21
			Total records = 288
ALL RECORDS = 452			
