# Supplementary material for: Influencing Cardiovascular Outcomes through Heart Rate Variability Modulation: A Systematic Review
Source: Diagnostics (Basel). 2021 Nov 25;11(12):2198. doi: 10.3390/diagnostics11122198 (PMC8700170; doi:10.3390/diagnostics11122198)
Supplement: Supplementary file 1 [file diagnostics-11-02198-s001.zip › diagnostics-1456342-supplementary/Table S2 Quality assessment.pdf]

Table S2. Quality assessment of included studies using Newcastle-Ottawa scale.

Study	Case definition	Case representativeness	Selection of controls	Definition of controls	Comparability	Exposure ascertainment	Ascertainment methods	Non-response rate	Total
Bernardi et al, 2002	*	*	*	*	*	*	*		7
Albuquerque Cacique et al, 2021	*	*	*	NA	NA	*	*		5
Joseph et al, 2005	*	*	*	*	*	*	*		7

NA = not applicable.

Good quality: 3 or 4 stars in selection domain AND 1 or 2 stars in comparability domain AND 2 or 3 stars in outcome/exposure domain. Fair quality: 2 stars in selection domain AND 1 or 2 stars in comparability domain AND 2 or 3 stars in outcome/exposure domain. Poor quality: 0 or 1 star in selection domain OR 0 stars in comparability domain OR 0 or 1 stars in outcome/exposure domain.
