# Supplementary figures and images for: Influencing Cardiovascular Outcomes through Heart Rate Variability Modulation: A Systematic Review
Source: Diagnostics (Basel). 2021 Nov 25;11(12):2198. doi: 10.3390/diagnostics11122198 (PMC8700170; doi:10.3390/diagnostics11122198)

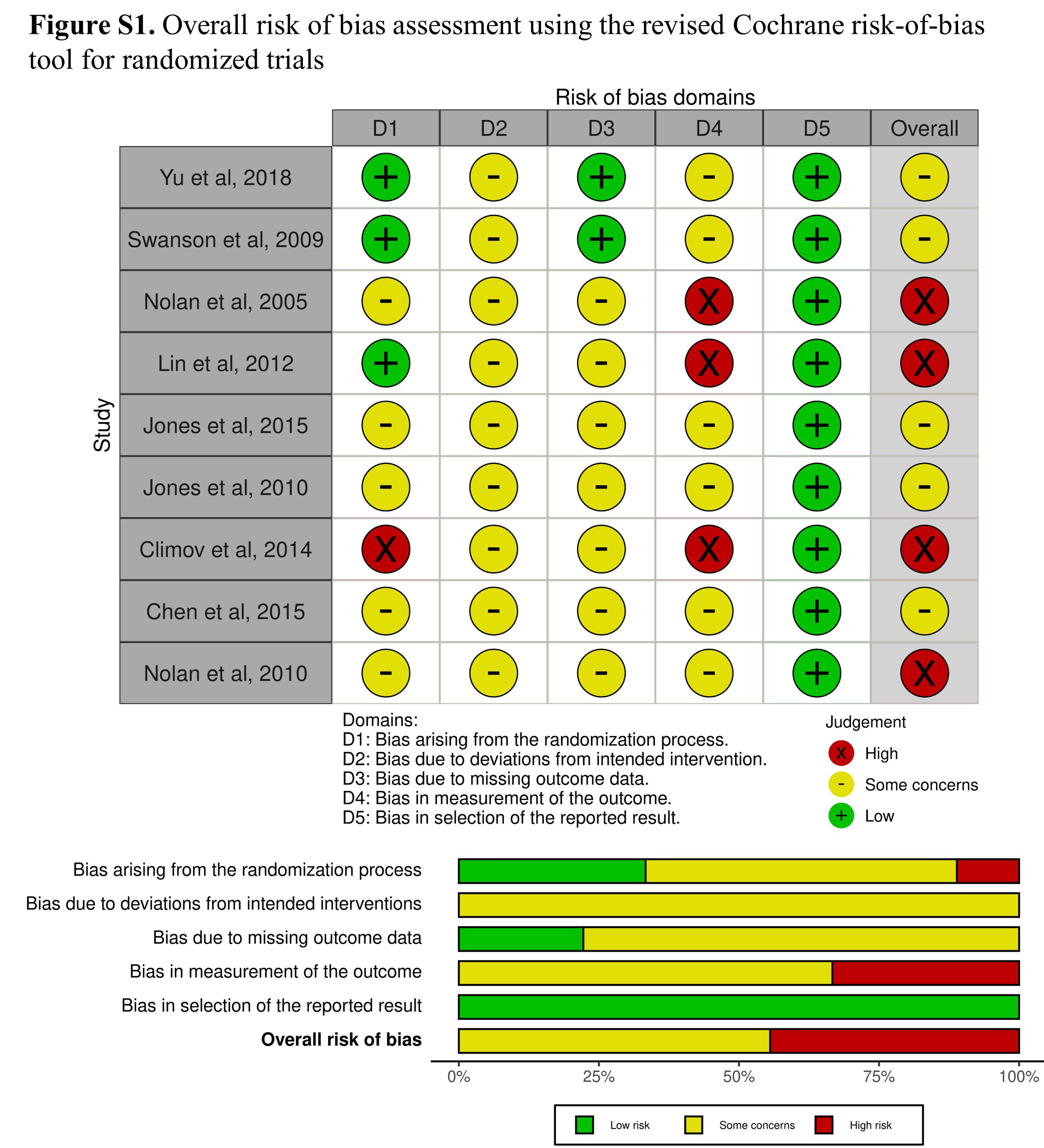

Supplement: Supplementary file 1 [file diagnostics-11-02198-s001.zip › diagnostics-1456342-supplementary/Figure S1. Risk of Bias.tif]
